# Evaluation of two commercial global miRNA expression profiling platforms for detection of less abundant miRNAs

**DOI:** 10.1186/1471-2164-12-435

**Published:** 2011-08-26

**Authors:** Steffen G Jensen, Philippe Lamy, Mads H Rasmussen, Marie S Ostenfeld, Lars Dyrskjøt, Torben F Ørntoft, Claus L Andersen

**Affiliations:** 1 Department of Molecular Medicine (MOMA), Aarhus University Hospital-Skejby, DK-8200 Aarhus N, Denmark

## Abstract

**Background:**

microRNAs (miRNA) are short, endogenous transcripts that negatively regulate the expression of specific mRNA targets. miRNAs are found both in tissues and body fluids such as plasma. A major perspective for the use of miRNAs in the clinical setting is as diagnostic plasma markers for neoplasia. While miRNAs are abundant in tissues, they are often scarce in plasma. For quantification of miRNA in plasma it is therefore of importance to use a platform with high sensitivity and linear performance in the low concentration range. This motivated us to evaluate the performance of three commonly used commercial miRNA quantification platforms: GeneChip miRNA 2.0 Array, miRCURY Ready-to-Use PCR, Human panel I+II V1.M, and TaqMan Human MicroRNA Array v3.0.

**Results:**

Using synthetic miRNA samples and plasma RNA samples spiked with different ratios of 174 synthetic miRNAs we assessed the performance characteristics reproducibility, recovery, specificity, sensitivity and linearity. It was found that while the qRT-PCR based platforms were sufficiently sensitive to reproducibly detect miRNAs at the abundance levels found in human plasma, the array based platform was not. At high miRNA levels both qRT-PCR based platforms performed well in terms of specificity, reproducibility and recovery. At low miRNA levels, as in plasma, the miRCURY platform showed better sensitivity and linearity than the TaqMan platform.

**Conclusion:**

For profiling clinical samples with low miRNA abundance, such as plasma samples, the miRCURY platform with its better sensitivity and linearity would probably be superior.

## Background

microRNAs (miRNAs) are short 20-23 nucleotide long non-coding RNAs that are widely distributed in almost all eukaryotic organisms. They have multiple functions however the main function is believed to be post transcriptional regulation of protein levels [1,2]. While miRNAs are often abundant in tissues, the amount found circulating in body fluids such as plasma and serum is often limited. It has been reported that the total RNA level in plasma is in the range 6-300 ng/ml [3,4] and that the miRNA fraction constitutes only a few percent of this [5]. The mechanisms regulating secretion of miRNA into circulation is still unclear. Reports have shown that while endogenous miRNAs appear stable in plasma/serum exogenous miRNAs are not, and as a result of this it has been suggested that endogenous circulating miRNAs are either encapsulated in microvesicles or bound to RNA-binding proteins in complexes, e.g. Ago2 and NPM1, protecting them from degradation [6-8]. Detailed knowledge of the biological function of circulating miRNA does not exist, however it has been shown that vesicular miRNAs can be transferred from cell to cell and influence the behavior of the recipient cells [9].

MicroRNAs have been reported deregulated in various diseases. Independent studies on different tissue materials have shown that miRNA expression profiles differ between healthy and diseased tissue, and various lines of evidence indicate that they have great potential as diagnostic, prognostic, and predictive biomarkers [10]. It is technically demanding to quantify mature miRNAs based on the often low-abundance, short length of mature miRNA, homology between miRNA species, and the inclusion of the mature miRNA sequence in the primary miRNA (pri-miRNA) and precursor miRNA (pre-miRNA) transcripts. The latter makes it difficult to construct assays that are specific for the mature form. Nevertheless, multiple platforms for quantifying mature miRNAs exist, which are most commonly based on either quantitative real-time PCR (qRT-PCR) or microarrays, although alternatives exist [11]. Common to all platforms are that a reverse transcription step is required to convert the RNA into cDNA prior to quantification. To date two different approaches have been utilized for this step. In the first approach, miRNAs are reverse transcribed individually using miRNA-specific reverse transcription primers e.g. stemloop primers. In the second approach, miRNAs are first tailed with a common sequence and then reverse transcribed using a universal primer. Subsequently, the cDNA levels of specific miRNAs are quantified by qPCR or microarray [11].

The qRT-PCR based platforms promise to be more sensitive than array based miRNA quantification platforms [12], and their use for analyzing samples with low miRNA levels, such as human plasma, is increasing [13-18]. A few reports have assessed the performance of a number of miRNA quantifying platforms, however they mostly focused on array based platforms and analysis of miRNA rich samples [12,19-21]. As one of the major perspectives for miRNAs in the clinical setting is the use as diagnostic markers in screening for neoplasia in body fluids, it is very important to evaluate platform performance at the low miRNA levels found in such samples.

This motivated us to evaluate the performance of three commonly used commercial miRNA quantification platforms: GeneChip miRNA 2.0 Array (Affymetrix), miRCURY Ready-to-Use PCR, Human panel I+II V1.M (Exiqon), and TaqMan^® ^Human MicroRNA Array v3.0 (Applied Biosystems Inc. (ABI)). Using plasma RNAs spiked with varying concentrations of 174 synthetic miRNAs we evaluated the specificity, accuracy, linearity, and sensitivity of the platforms.

## Methods

### Patients/Plasma/RNA isolation

The research was conducted in accordance with the Helsinki Declaration. Informed written consent was obtained from all patients according to local ethical regulations, and research protocols were approved by the Central Denmark Region Committees on Biomedical Research Ethics (J. no. 1999/4678). One milliliter of plasma was isolated from each of seven blood samples drawn prior to surgery from patients diagnosed with Colorectal Cancer (CRC). The plasma was pooled and total RNA including small RNAs, were purified according to the protocol supplied with the miRNeasy Mini Kit (Qiagen) with the exception that 1 μg MS2 carrier RNA (Roche) was added to the QIAzol Lysis Reagent prior to RNA purification in order to maximize the yield and minimize purification efficiency variation.

### Synthetic miRNA samples and plasma RNA samples spiked with synthetic miRNAs

Two pools of 88 (pool A) and 86 (pool B) synthetic miRNAs with sequences corresponding to miRBase v14 were generated (Additional file 1, Table S1). Each pool contained 10^8 ^copies of each miRNA/μl dissolved in RNA storage solution buffer (Ambion) supplemented with MS2 carrier in a final concentration 10 ng/μl. These pools were mixed in ratios 1:4 and 4:1 to formulate the two synthetic samples #1 and #2. Synthetic sample #1 contained 0.2*10^7 ^copies/μl of the pool A miRNAs and 0.8*10^7 ^copies/μl of the pool B miRNAs and vice versa for the synthetic sample #2. Hence, comparison of miRNA quantifications from the two synthetic samples should show four-fold differences for all miRNAs.

To enable evaluation of platform performance on complex samples the synthetic miRNAs were spiked into two RNA aliquots from the pooled plasma RNA preparation described above. Thereby, two novel spiked plasma RNA samples (spiked plasma RNA #1 and #2) having the same concentrations of the synthetic miRNAs as the synthetic samples were generated.

### Mapping of platform assays to miRBase v14 and identification of assays targeting the synthetic miRNAs

Complete lists of assays (including the miRBase version used for assay design) on the GeneChip miRNA 2.0 Array (Affymetrix), the miRCURY Ready-to-Use PCR V1.M panels (Exiqon), and the TaqMan^® ^Human MicroRNA Array v3.0 TLDA cards (ABI) were obtained from the manufacturers. All platforms included various assays targeting sequences that could not be mapped to miRBase v14 or targeted non-human sequences. These were excluded from further analysis. In order to identify assays targeting the synthetic miRNAs, the assay target sequences were matched to the list of synthetic miRNAs. This revealed that of the 174 synthetic miRNAs the GeneChip platform contained matching assays for all 174, the miRCURY platform for 143, and the TaqMan platform for 155. In total, 125 of the synthetic miRNAs were represented on all three platforms (Additional file 1, Table S1).

### Identification of assays targeting miRNAs with sequence homology to one or more of the synthetic miRNAs

The assay target sequences were aligned to the sequences of the synthetic miRNAs using a Smith-Waterman based sequence alignment algorithm, allowing up to four mismatches and eight nucleotide overhangs. Consequently, we identified assays targeting miRNAs with sequence homology to one or more of the synthetic miRNAs. The alignments are supplied in Additional file 2, Table S2.

### RNA input for cDNA synthesis

When cDNA for the individual platforms were generated from the synthetic and spiked plasma RNA samples, the sample input quantities were adjusted such that, unless stated otherwise, the same number of synthetic miRNA copies were added per cDNA reaction (1*10^6 ^and 0.25*10^6 ^copies for the high- and low-abundance synthetic miRNAs). Similarly, when cDNA was generated from the pure plasma sample (including the no-RT control) the same volume of plasma RNA as for the spiked-plasma RNA samples was used (corresponding to 1/6 of the RNA extracted from 250 μl plasma). By this approach we enabled direct comparison of the miRNA quantities estimated from the cDNAs.

Duplicate aliquots of all investigated RNA samples were used for two separate reverse transcription reactions and the products of each reaction was used in separate qPCR amplifications. This enabled evaluation of reproducibility, of the reverse transcription and subsequent qPCR amplifications.

### GeneChip assay setup

RNA was labeled using the 3DNA Array Detection Flash Tag RNA Labeling Kit (Genishere), according to manufacturers recommendations. First, poly(A) tailing was carried out at 37°C for 15 min in a volume of 15 μl reaction mix, which contains 1× Reaction Buffer, 1.5 μl 25 mM MnCl2, 1 μl 1:500 diluted ATP Mix and 1 μl PAP enzyme. Second, FlashTag Ligation was performed at room temperature for 30 min by adding 4 μl of 5× FlashTag Ligation Mix Biotin and 2 μl T4 DNA Ligase into the 15 μl of reaction mix. 2.5 μl of Stop Solution was added to stop the reaction. Samples were hybridized, washed and scanned with an Affymetrix Scanner.

### miRCURY LNA™ Universal RT microRNA PCR setup

cDNA synthesis and real-time qPCR was performed using the miRCURY LNA™ Universal RT microRNA PCR system (Exiqon, Denmark) according to the manufacturers instructions. In brief, the RNA were tailed with a poly(A) sequence at their 3'end and then reverse transcribed into cDNA using a universal poly(T) primer with a 3'end degenerate anchor and a 5'end universal tag. The cDNA products were subsequently diluted 125 fold and transferred to the Ready-to-use microRNA PCR Human Panels (I + II) and quantified using SYBR green based real time PCR and LNA enhanced miRNA specific primers. The qPCRs were run on a 7900HT thermocycler (ABI) using the thermal-cycling parameters recommended by Exiqon. Raw Ct values were calculated as recommended by Exiqon using the RQ manager software v1.2.1 (ABI) with manual settings for threshold and baseline, i.e. all miRCURY assays were analyzed using a ΔRn threshold of 60 and baseline subtraction using cycles 1-14.

### TaqMan^® ^Human MicroRNA Array Set v3.0 setup

cDNA synthesis, pre-amplification, and real-time qPCR was performed as described in the protocol associated with the TaqMan^® ^Human MicroRNA Arrays Set v3.0 (ABI). In brief, RNA was reverse transcribed using Megaplex RT Stemloop primers (pool A or B) and the TaqMan miRNA reverse transcription kit. For optimal sensitivity ABI recommends inclusion of a pre-amplification step. In this step the product of the reverse transcription reaction was pre-amplified using Megaplex PreAmp primers (pool A or B) and TaqMan PreAmp Master Mix. Finally, the pre-amplification product was diluted as indicated by the manufacturer and loaded onto the TaqMan A or B Array. The arrays were run using a 7900HT thermocycler (ABI). Raw Ct values were calculated as recommended by ABI using the RQ manager software v1.2.1 (ABI) with automatic baseline and threshold settings.

### Statistical analysis and sequence alignment

Variance comparison test, Poisson randomness test, Mann-Whitney U test or Fisher's exact test were applied to assess differences or proportions in the obtained data [22]. P-values < 0.05 were considered significant. Reproducibility and linearity were evaluated using Pearson correlation coefficients. The statistical analyses were carried out using STATA v10.1 (Statacorp), Excel 2007 (Microsoft), or the open source R-software http://www.r-project.org/. Sequence alignments were performed using a Smith-Waterman based sequence alignment algorithm [23].

## Results

In a pilot study evaluating the amount of plasma RNA input necessary to detect miRNAs we found that the qRT-PCR based platforms (TaqMan from ABI and miRCURY from Exiqon) reproducibly detected miRNAs using inputs ranging from all the RNA extracted from 250 μl plasma down to as little as 1/100 of this input (data not shown). In order not to assess the platforms at the brink of their sensitivity it was decided that for the platform evaluation the input per cDNA synthesis should be 1/6 of the RNA from 250 μl plasma. The GeneChip miRNA 2.0 Array platform from Affymetrix repeatedly failed to produce reliable signals at this input level (data not shown). This was not surprising, as the total amount of RNA isolated from 250 μl plasma was well below the minimum requirement of 100 ng total RNA stated in the manual for the platform. Consequently, due to the limited sensitivity the GeneChip platform was excluded from further assessment.

### Specificity

**Figure 1:**
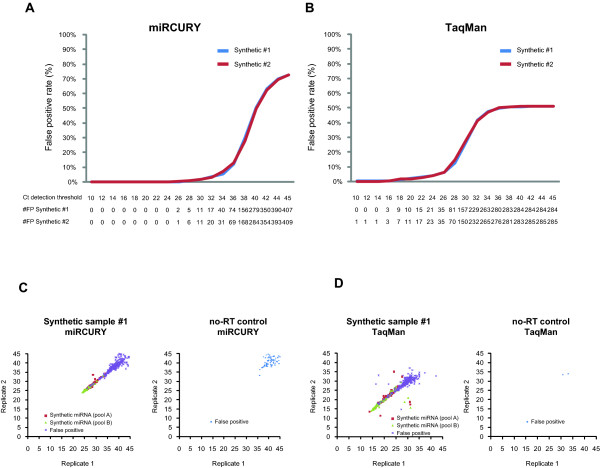
**Assessment of the assay specificities of the miRCURY and TaqMan platforms**. Utilizing the two synthetic samples #1 and #2 with known miRNA contents enabled us to investigate the false positive rate of the individual platforms at given Ct detection thresholds. The rates for the individual platforms are provided in (A) miRCURY and (B) TaqMan. Clearly, the number of false positives increased with increasing Ct detection threshold indicating that carefully chosen Ct detection thresholds could potentially reduce the number of false positives without affecting the true positives. In order to determine the thresholds for the two platforms the raw Ct's of the synthetic samples were compared to no-RT controls. As illustrated by plotting the raw Ct's for the duplicate measurements of the synthetic sample #1 and the no-RT-control for miRCURY (C) and TaqMan (D) thresholds set at Ct = 38 and Ct = 30, respectively would dramatically reduce the number of false positives. #FP, number of false positives.

To enable assessment of specificity and recovery two synthetic samples with known quantities of 174 synthetic miRNAs were formulated. The false positive rate (false positives/(false positives + true negatives)), which equals 1-specificity, was calculated for a range of Ct detection thresholds. In other words it was investigated how many assays yielded a signal at a given Ct detection threshold despite the target miRNA not being present in the synthetic sample (Figure 1A and 1B). Both platforms yielded false positives. Consistent with the pre-amplification step included in the TaqMan setup false positives were detected at lower Ct values for this platform than for the miRCURY platform in which a pre-amplification step is not included. With the pre-amplification difference in mind the false positive rates of the two platforms were similar (Figure 1A and 1B). For both platforms it was found that the number of false positives increased with increasing detection thresholds. Noticeable, the majority of the false positives were detected at higher Ct values than the true positives (Figure 1C and 1D, left panels) indicating that applying carefully chosen Ct detection thresholds is a reasonable approach to ensure acceptable false discovery rates. To help defining these thresholds "no reverse transcription" controls were performed (Figure 1C and 1D, right panels). This showed that while the TaqMan platform generally did not produce signals in a no-RT setting, a subset of the miRCURY assays (on average 66 of the 689 assays on the platform) did so at late Ct values (generally above cycle 38). A Ct detection threshold of 38 was therefore implemented for the miRCURY platform, corresponding to a false positive rate of ~30% in the synthetic samples (Figure 1A). Aiming at similar false positive rate the Ct detection threshold for the TaqMan platform was set to 30 (Figure 1B). If not mentioned otherwise, these threshold cycles were used throughout the study.

**Figure 2:**
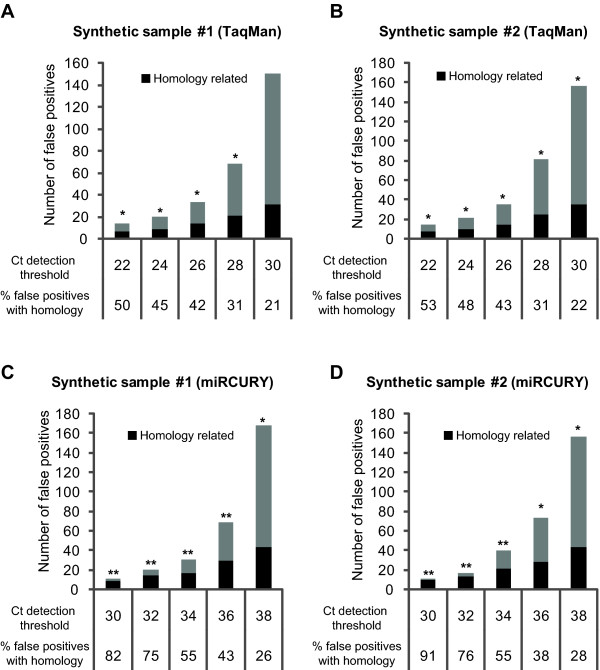
**Assessment of the platforms ability to discriminate miRNAs with close sequence homology**. Using the synthetic samples, with known miRNA content, it was investigated how many of the false positives detections (miRNAs not in the sample) at a given Ct detection threshold could be related to sequence homology to a miRNA in the sample. Plotted are the number of false positives detected in the synthetic samples at given Ct detection thresholds and it is indicated how many of these can be related to sequence homology. (A, B) TaqMan analysis of synthetic samples #1 and #2. (C, D) miRCURY analysis of synthetic samples #1 and #2. A Poisson randomness test was used to evaluate if the fraction of homology related false positives at a given Ct detection threshold was significantly higher than expected. The expected fraction was defined as the number of potential false positives (the number of assays on a given platform targeting miRNAs not present in the investigated sample) that have sequence homology to miRNAs in the sample. As the synthetic samples #1 and #2 contain the same miRNAs (but in different concentrations) the expected fraction is the same for the two samples. For the TaqMan platform the expected fraction is 93 out of 565 (16%) miRNAs and for the miRCURY platform it is 102 out of 552 (18%). **p < 0.0001, *p < 0.05.

Next we reasoned that a fraction of the false positives could be caused by assay cross-reaction associated with the close sequence relationship between some mature miRNAs differing with as little as a single nucleotide for some miRNAs. To assess the impact of this issue we examined how many of the false positives showed homology (a nucleotide difference of 4 or less) to one of the 174 synthetic miRNAs. This analysis showed for both platforms that the fraction of homology related false positives was at its highest at low Ct's and gradually decreased with increasing Ct detection thresholds. Stressing the importance of homology as a cause of false positives, we found that the homology related false positives were significantly enriched (p < 0.05, Poisson randomness test) at nearly all Ct detection thresholds for both platforms (Figure 2). While homology causes false positives for both platforms the extent was different, i.e. at low Ct's nearly all the false positives of the miRCURY platform was attributable to homology (80-90%), whilst for the TaqMan platform the equivalent number was only ~50-53%. This indicates that for this platform other causes also play a considerable role in the formation of false positives in the low Ct range (Figure 2). Our analysis further demonstrated, for both platforms, that the fewer the number of mismatches, the higher the tendency for a homologous assay to cross-react and cause a false positive detection (Table 1).

**Table 1:** The number of mismatches to homologous synthetic miRNAs impacts assay cross-reaction tendency¤.

	miRCURY					TaqMan				
	
Number of nucleotide mismatches to synthetic miRNA*	0	1	2	3	4	0	1	2	3	4
Number of assays§	3	18	34	52	60	3	18	30	45	57
Number of false positive assays (n)#	3	17	25	38	40	2	15	22	26	33
False positive rate (false positives/number of assays) (%)	100	94	74	73	67	67	83	73	58	58

¤ A given assay can be homologous to more than one synthetic miRNA and thereby appear in more than one of the mismatch categories. This may cause the false positive rates to be over estimated.

* 0 mismatches means that only the length differs between the synthetic miRNA and the miRNA assayed.

§Number of assays with the given number of nucleotide mismatches to a miRNA in the synthetic samples.

#An assay was scored as false positive if detected at Ct < 38 (miRCURY) or Ct < 30 (TaqMan) in one or more of the synthetic samples #1 and #2.

### Recovery

**Figure 3:**
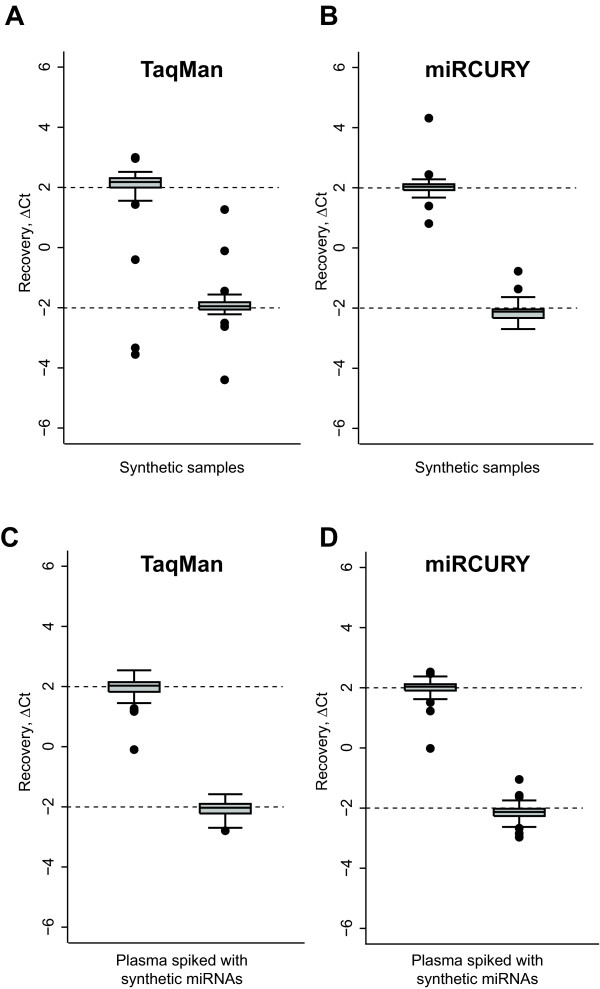
**Assessment of the capability of the platforms to recover known miRNA copy number differences**. The ability of the platforms to recover known four-fold miRNA copy number differences between samples was assessed by calculating ΔCt's for 125 synthetic miRNAs common to both the TaqMan and miRCURY platforms. Shown are boxplots of the ΔCt's obtained for TaqMan (A, C) and miRCURY (B, D). Recovery was assessed both in the synthetic samples i.e. consisting solely of synthetic miRNAs (A, B) and the plasma RNA samples spiked with synthetic miRNAs (C, D). Separate plots were made for miRNAs that were four fold higher (ΔCt = 2) and lower (ΔCt = -2) in sample 1 compared to 2.

The synthetic samples #1 and #2 were constructed such that they contained the exact same 174 miRNAs but in different concentrations. For 88 of the miRNAs the number of transcript copies was four-fold lower in synthetic sample #1 than #2 and for the remaining 86 miRNAs it was four-fold higher. The ability of the platforms to recover these known miRNA quantity differences was assessed for each miRNA by calculating the difference in Ct values between the two samples. As duplicate cDNA syntheses and qPCR setups were performed for all samples investigated, the reported differences were in practice calculated as the difference between the averages of the duplicates. For consistency of the recovery analysis, assays were excluded if one or more of the measurements were detected after the detection threshold. In order to ease comparison of the platforms the analysis was restricted to the 125 of the 174 synthetic miRNAs for which both platforms contained assays. The recovered fold-changes of both platforms were close to the expected +/-2 Ct (four-fold copy number differences). In the following the miRNAs expected to yield +/-2 Ct differences, respectively are reported separately. For the TaqMan platform only 109 of the 125 assays were detected in all measurements. The number of included assays, median, interquartile range (iqt), and variance for the miRNAs with an expected +2Ct difference were (56, 2.18, 0.30, 0.19) and for the miRNAs with an expected -2 Ct difference they were (53, -1.97 0.19, 0.35). The miRCURY platform detected all 125 assays and for the miRNAs with expected +2Ct and -2Ct differences the number of included assays, median, iqt, and variance were (63, 2.03, 0.20, 0.14) and (62, -2.04, 0.29, 0.09), respectively (Figure 3A and 3B). The median fold changes recovered by the two platforms were not significantly different (p-values all > 0.65, Mann-Whitney U test). However, the variance of the recovered fold-changes was significantly larger for the TaqMan platform than the miRCURY platform (p < 0.001, variance comparison test)) indicating a poorer recovery accuracy of the TaqMan platform. Notably, this result was obtained even though the analysis of the TaqMan platform included only 109 of 125 common miRNAs and the analysis of the miRCURY platform included them all. The primary reason for the lower number of included assays for the TaqMan platform, and likely also for the larger variance of the recovered fold changes, was individual wells amplifying poorly during qPCR in one of the TaqMan replicates (Figure 1D left panel and Additional file 3, Figure S1) generating outliers often detected beyond the Ct detection threshold causing exclusion of the assays. The basis of these poor amplifications remains unknown, but since the same assays amplified satisfactorily with other samples these outliers may be related to the particular TaqMan arrays used for the synthetic samples (Additional file 3, Figure S1).

Next, recovery performance was assessed in samples with biological level complexity, i.e. plasma-derived RNA with the synthetic miRNAs spiked-in. Two aliquots of the same plasma RNA preparation were spiked with synthetic samples #1 and #2, generating spiked plasma RNA samples #1 and #2. The TaqMan and miRCURY platforms identified 99 and 114 endogenous miRNAs in the non-spiked plasma, respectively (Table 2). Theoretically, these endogenous miRNAs should not impact the platforms ability to recover the expected miRNA quantity differences between the spiked plasma samples. Our analyses also confirmed this to be the case with median recoveries very close to the expected +/-2 Ct for both platforms. The variances of the fold changes recovered by the two platforms were not significantly different (p = 0.92, variance comparison test), indicating that in the spiked plasma samples the recovery accuracy of the two platforms were equal. The number of assays included, median, iqt, and variance for the TaqMan platform were (62, 2.03, 3.32, 0.15) and (57, -2.03, 0.32, 0.06) and for the miRCURY platform (62, 2.04, 0.21, 0.11) and (61, -2.13, 0.24, 0.09) (Figure 3C and 3D). It is noticeable that the number of included TaqMan assays in this analysis nearly reached the level of miRCURY assays (119 vs. 123 out 125 assays) indicating that the outliers observed for the TaqMan platform with the synthetic samples were due to the specific LDA cards used rather than general poor performance of the platform.

**Table 2:** Assessment of platform reproducibility, Pearson correlation of duplicate measurements§.

Sample	All assays detected		The 125 spike-in miRNAs for which both platforms have assays	
	miRCURY	TaqMan	miRCURY	TaqMan
	r (n detected)	r (n detected)	r (n detected)	r (n detected)
Synthetic #1	0.991 (253)	0.936 (233)	0.968 (125)	0.947 (109)
Synthetic #2	0.993 (244)	0.892 (259)	0.988 (125)	0.886 (116)
Spiked plasma RNA #1	0.994 (309)	0.983 (308)	0.995 (124)	0.982 (123)
Spiked plasma RNA #2	0.992 (300)	0.989 (308)	0.990 (124)	0.989 (119)
Plasma	0.954 (114)	0.959 (99)	na	na
No-RT control	0.773 (8)	na (0)	na	na

§ Calculated for all assays with Ct's below the detection threshold (TaqMan Ct = 30, miRCURY Ct = 38) in both duplicates.

Having revealed that both platforms have difficulties recovering the expected fold change for a small subset of miRNAs we speculated if these poorly recovered miRNAs were the same for both platforms. This was assessed by testing if the differences (observed - expected fold change) of the two platforms were correlated. The analysis found no correlations in neither the synthetic nor the spiked plasma samples (r~0, Pearson correlation coefficient) (Additional file 3, Figure S2), indicating that the problematic miRNAs are different for the two platforms. Altogether, the recovery assessment indicates that the majority of the assays on both platforms are capable of detecting four-fold copy number differences and that the miRCURY platform appears to perform slightly better than the TaqMan platform. Further experiments are needed to evaluate if recovery is equally good at fold changes less than four-fold, however this is beyond the scope of the present study.

### Reproducibility

Many processing steps, each potentially introducing variation, are required to quantify miRNA transcript levels in biological samples, including plasma. To enable evaluation of reproducibility duplicate aliquots of all investigated RNA samples were used for two separate reverse transcription reactions and the products of each reaction was used in separate qPCR amplifications. A total of five RNA samples were investigated: synthetic RNA samples #1 and #2, spiked plasma RNA samples #1 and #2 and pure plasma RNA. The comparison of every duplicate pairs demonstrated median Pearson correlation coefficients of 0.985 and 0.952 for the miRCURY and TaqMan platforms, respectively (Table 2). As already mentioned, a few outlier assays with low reproducibility were observed for the TaqMan platform in the analysis of the synthetic samples (Additional file 3, Figure S1). Since the assays performed otherwise successfully this may relate to the specific LDA cards used for the synthetic samples rather than to the assays themselves. To facilitate comparison of the reproducibility measures for the two platforms, the analysis was repeated and this time restricted to the 125 spike-in miRNAs for which assays were present on both platforms. The same pattern was observed indicating that data produced by the miRCURY platform was slightly more reproducible than the TaqMan platform (Table 2).

### Sensitivity and linearity

To enable assessment of sensitivity and linearity of the platforms, a five point 10-fold dilution series of spiked plasma RNA sample #1 were generated. For the miRCURY platform this translates to 5000/20000 (pool A/pool B miRNAs), 500/2000, 50/200, 5/20, and 0.5/2 template copies added per final PCR amplification at each of the five dilution points assuming that all the pre-PCR steps applied were 100% efficient. Even though the same RNA inputs were also used for the TaqMan cDNA syntheses, similar estimates of the input copy numbers for the final PCRs cannot be calculated as the TaqMan cDNA was pre-amplified.

Like for the other samples investigated in this study duplicate aliquots of each dilution point were used for two separate reverse transcription reactions and qPCR amplifications. This enabled analysis of how decreasing input material impacted reproducibility. In order to ease inter-platform comparison, the analysis was again restricted to the 125 miRNAs assayed by both platforms. As expected, the number of detected miRNAs and the reproducibility of the measurements decreased with increasing dilution for both platforms (Table 3). Neither of the platforms were sensitive enough to reproducibly detect miRNAs at the final dilution point (0.5/2 template copies per PCR amplification) (Table 3). At the dilution points with the largest input concentrations the platforms performed similarly, but at the dilution points with 5/20 and 50/200 template copies per PCR amplification the sensitivity of the TaqMan platform decreased significantly (p ≤ 0.001, Fishers Exact test) compared to the miRCURY platform (Table 3). At 5/20 template copies per PCR amplification the miRCURY platform detected 74% (93/125) of the miRNAs, while the TaqMan platform only 47% (59/125). Noticeably, the reproducibility of the miRCURY platform remained at the same level as the TaqMan platform despite detecting 50% more miRNAs.

**Table 3:** Assessment of sensitivity and reproducibility at different miRNA input levels, spanning five orders of magnitude, Pearson correlation of duplicate measurements§.

	The 125 spike-in miRNAs for which both platforms have assays	
	miRCURY	TaqMan
miRNA copies pr. PCR amplification	r (n detected)	r (n detected)
5,000/20,000	0.995 (124)	0.981 (123)
500/2,000	0.901 (124)	0.853 (121)
		0.990 (116)*
50/200	0.920 (119)	0.930 (101)
5/20	0.829 (93)	0.843 (59)
0.5/2	0.228 (35)	0.249 (34)

§ Calculated for all assays detected in both duplicates.

* Calculated after removing five assays with outlier measurements in one of the duplicates.

As noticed during the analysis of the synthetic samples, the TaqMan platform had a few outliers (n = 5) in the duplicate measurements in the second dilution point. Again, these appeared to be caused by a few individual wells in one of the LDA cards amplifying poorly (data not shown). When these assays were excluded the Pearson correlation increased from 0.85 to 0.99 (Table 3), indicating that the TaqMan platform at this input level generates highly reproducible measurements.

**Figure 4:**
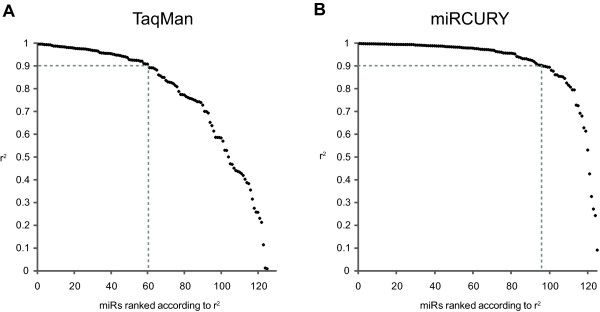
**Evaluation of assay linearity at low miRNA input levels for 125 spike-in miRNAs common to the TaqMan and miRCURY platforms**. Linearity was assessed based on a four point dilution series, ranging four orders of magnitude, of the spiked plasma RNA sample #1. For ease of platform comparison the data presented was restricted to the 125 spiked miRNAs queried by both platforms. The template input per PCR reaction of these 125 miRNAs ranged from 5 - 5,000 template copies (n = 62) and 20-20,000 (n = 63). Each dilution point was measured in duplicate and the linearity of each of the 125 assays was estimated by calculating the squared Pearson correlation coefficient, r^2 ^of these measurements. Plotted are the obtained r^2 ^values for (A) the TaqMan platform and (B) the miRCURY platform. Dashed lines correspond to number of assays with r^2 ^≥ 0.9.

Next we assessed the linearity of the platforms by calculating Pearson correlations for each assay across the dilution series. As neither of the platforms produced acceptable measurements at the lowest input level of the dilution series this point was excluded. For the linearity analysis no detection thresholds were applied and all measurements classified as "undetermined" were replaced by the Ct threshold values 30 (TaqMan) or 38 (miRCURY) corresponding to background level. This replacement was necessary to enable assessment of linearity across all four dilution points for all 125 assays. The analysis showed that the number of assays with a linear performance (r^2 ^≥ 0.9) across the four log scales was significantly higher for the miRCURY platform than for the TaqMan platform (p < 0.001, Fisher's Exact test) with 77% (96/125) of the miRCURY assays and only 48% (60/125) of the TaqMan assays having a r^2 ^≥ 0.9 (Figure 4).

## Discussion

This study reports the results of an evaluation of the performance of three commonly used commercially available miRNA quantification platforms. The focus was particular on performance in relation to minute levels RNA input, i.e. in the range of what can be extracted from 250 μl of human plasma. Consistent with previous reports we found qRT-PCR based platforms to have higher sensitivity than microarray based platforms [12]. In fact in our hands the GeneChip miRNA 2.0 platform was not sensitive enough to reliably produce signals with the plasma RNA input levels studied. We nevertheless continued assessing the two remaining qRT-PCR platforms.

Using samples with known miRNA contents we were able to show that at abundant miRNA levels the technical reproducibility and sensitivity of these platforms was good and comparable. However, at low miRNA levels, particularly at 50/200 copies or below, the sensitivity of the miRCURY platform was significantly higher than for the TaqMan platform. Consistent with this we found that the number of miRCURY assays with a high degree of linearity (r^2 ^≥ 0.9) across four log scales of miRNA copies was significantly higher than for the TaqMan platform. Our evaluation of the two platforms' ability to recover four-fold differences revealed that at the investigated miRNA concentrations both platforms have an acceptable recovery. However, based on the better sensitivity and linearity of the miRCURY platform it is likely that at lower miRNA concentrations the results of a similar recovery analysis would have been in favor of the miRCURY platform. We did not address recovery of fold-changes less than four-fold, but expect that the performance of both platforms will decline with decreasing fold-changes.

Considering the inclusion of the reported sensitivity improving pre-amplification step in the TaqMan protocol it was surprising to find that at low miRNA levels the sensitivity and linearity of the miRCURY platform was better than the TaqMan platform. This could indicate that the sensitivity boosting effect of the pre-amplification step is less prominent than reported [24]. However, it should be noted that other differences between the platforms also exist, and alternatively, these could also play a role for the better sensitivity of the miRCURY platform. For example, for cDNA synthesis the miRCURY platform uses a universal approach with poly(A) end-tailing and oligo(dT) primed reverse transcription, while the TaqMan approach megaplexes > 300 miRNA specific stem-loop primers for initiation of reverse transcription. Potentially the universal approach may be more robust and sensitive than the megaplexed approach. Another possibility is the inclusion of LNAs in the miRCURY primer designs. LNAs make assay design nearly independent of miRNA GC content and compensates for many of the compromises one otherwise would have to make with a short miRNA target sequence of just ~22 nucleotides. Noticeably, It has previously been reported that inclusion of LNAs improves both PCR specificity and sensitivity [25].

We also assessed the specificity of the platforms and strikingly noticed that while the TaqMan platform generally showed no false positives in the no-RT control nearly 10% of the miRCURY assays were positive (even though detected at late Ct's, > 38). This could indicate that the miRCURY platform is less specific than the TaqMan platform. However, our analyses of the synthetic samples revealed that within the operative range, i.e. from the first detection to the detection threshold, the two platforms produce nearly identical numbers and rates of false positives (Figure 1). Hence, for practical purposes the specificity of the two platforms appears equal. Importantly, the number of false positives produced by both platforms increased exponentially with increasing detection thresholds. Hence, for both platforms the detection threshold should be chosen with great care to obtain acceptable false discovery rates when profiling biological samples. Sequence similarity between miRNAs has been reported to be potential cause of false positive detections [11,26]. Consistent with this our analyses indicated that a significant fraction of the false positives, for both platforms, in particular at low Ct's was related to sequence homology (Figure 2). The latter is critical as this is also the detection range of the true positives making it practically impossible to distinguish the false from the true positives. As expected we found an inverse relationship between the number of nucleotide mismatches and the likelihood of a false positive detection (Table 1). While this was observed for both platforms the relationship was more pronounced for the miRCURY than the TaqMan platform. Along the same line the fraction of non-homology related false positives was larger for the TaqMan than the miRCURY platform (Figure 2). We do not understand the basis of the non-homology related false positives, but we perceive non-homology related amplification as more unspecific than homology related. In summary, our specificity evaluations indicate that the LNA based miRCURY platform compared to the stem-loop based TaqMan platform performs poorer in no-RT controls and slightly better, in terms of sequence specificity, in template containing samples. With both platforms it seems prudent to keep homology in mind when interpreting data from clinical samples and it may be advisable to consider carefully the likelihood of assay cross-reaction before taking a particular miRNA further, e.g. to *in vitro *studies.

We do not find it likely that the specificity issues of qRT-PCR will ever be completely eliminated; however, considering the ongoing technological shift to small RNA sequencing the issue may also soon be alleviated. The advantage of sequencing is that it is not hindered by variability in melting temperatures, coexpression of nearly identical miRNA family members, or post-transcriptional modifications. Moreover, it enables identification of novel miRNAs and unlike qRT-PCR the validity of sequencing results are not sensitive to changes in the miRNA sequences registered in miRBase. Currently the minimal input requirements for most small RNA liberary preparations are limiting for the use of the technology for profiling low abundance miRNA samples, such as human plasma. However, multiple approaches are being pursued to bring input requirements down, and the results are promising [27]. Other limitations include the RNA ligation and the PCR amplification steps in the library construction protocols both of which bear inherent biases [21]. Furthermore the tools for computational analysis of next generation sequencing data are still in their infancy. However, the technology has the potential to replace qRT-PCR as the preferred tool for profiling low abundance miRNA samples.

For now though, our data indicate that of the three tested platforms, the miRCURY platform with its better sensitivity and linearity in the low miRNA concentration range should be the platform of choice for analysis of low abundance miRNA samples. For analysis of samples with high miRNA abundance both qRT-PCR-platforms can be used, and probably also the GeneChip platform. However, when choosing platform one should keep in mind the poorly amplifying well phenomena we observed for some samples with the TaqMan platform. It caused artificial outlier measurements in the affected wells and while these outliers relatively easy can be identified by replication this approach may be prohibited by the availability of sample material and/or funding.

## Conclusion

For the analysis of samples with abundant miRNAs - as can often readily be obtained from most tissue samples - both the TaqMan and miRCURY platforms will most likely yield good results. However, for studying samples with low miRNA levels, such as plasma, our data indicate that it probably would be beneficial to use the miRCURY platform due to its better sensitivity and linearity in the low miRNA concentration range. Future application of the platforms on plasma from e.g. cancer patients will clarify whether miRNAs form a new reproducible family of molecules to be used for cancer diagnosis and follow-up.

## List of abbreviations

(ABI): Applied Biosystems Incorporated; (Ago2): Argonaute 2; (CRC): Colorectal Cancer; (cDNA): complementary DeoxyriboNucleic Acid; (Ct): Cycle threshold; (GC content): Guanine and Cytosine content; (Iqt): Inter quartile range; (LNA): Locked Nucleic Acid; (LDA): Low Density Array; (miRNA): microRiboNucleic Acid; (Nt): Nucleotide; (pre-miRNA): precursor microRiboNucleic Acid; (pri-miRNA): primary microRiboNucleic Acid; (qPCR): quantitative Polymerase Chain Reaction; (qRT-PCR): quantitative Reverse Transcription-Polymerase Chain Reaction; (RT): Reverse Transcription; (RNA): RiboNucleic Acid.

## Authors' contributions

SGJ carried out the experiments, analyzed the data, and helped draft the manuscript. PL assisted with the statistical analyses. MHR participated in the data analysis and critically revised the manuscript. LD, MSO, TFO and CLA conceived the study, and participated in its design and revision of the manuscript. CLA further coordinated the project, analyzed the data, and drafted the manuscript. All authors read and approved the final manuscript.

## Supplementary Material

Additional file 1 **Table S1: Synthetic miRNAs and associated assays on the GeneChip, miRCURY, and TaqMan platforms**. A list of the 174 synthetic miRNAs in the samples and the associated assays of the three investigated platforms. Click here for file

Additional file 2 **Table S2A: miRNA assays on the TaqMan platform with homology to the synthetic miRNAs from pool A or B**. Table S2B: miRNA assays on the miRCURY platform with homology to the synthetic miRNAs from pool A or B. Lists of assays on the TaqMan and miRCURY platforms with homology (≤ 4 nucleotides difference) to at least one of the synthetic miRNAs in the samples. Click here for file

Additional file 3 **Figure S1 Assessment of reproducibility by scatter plots of the Ct values from replica experiments**. Figure S2 Evaluation of whether the poorly recovered miRNAs of the TaqMan and miRCURY platforms were the same. The file contains the Figures S1 and S2. Click here for file
