# Supplementary material for: Evaluation of two commercial global miRNA expression profiling platforms for detection of less abundant miRNAs
Source: BMC Genomics. 2011 Aug 26;12:435. doi: 10.1186/1471-2164-12-435 (PMC3184117; doi:10.1186/1471-2164-12-435)
Supplement: Additional file 3 — Figure S1 Assessment of reproducibility by scatter plots of the Ct values from replica experiments. Figure S2 Evaluation of whether the poorly recovered miRNAs of the TaqMan and miRCURY platforms were the same. The file contains the Figures S1 and S2. [file 1471-2164-12-435-S3.PDF]

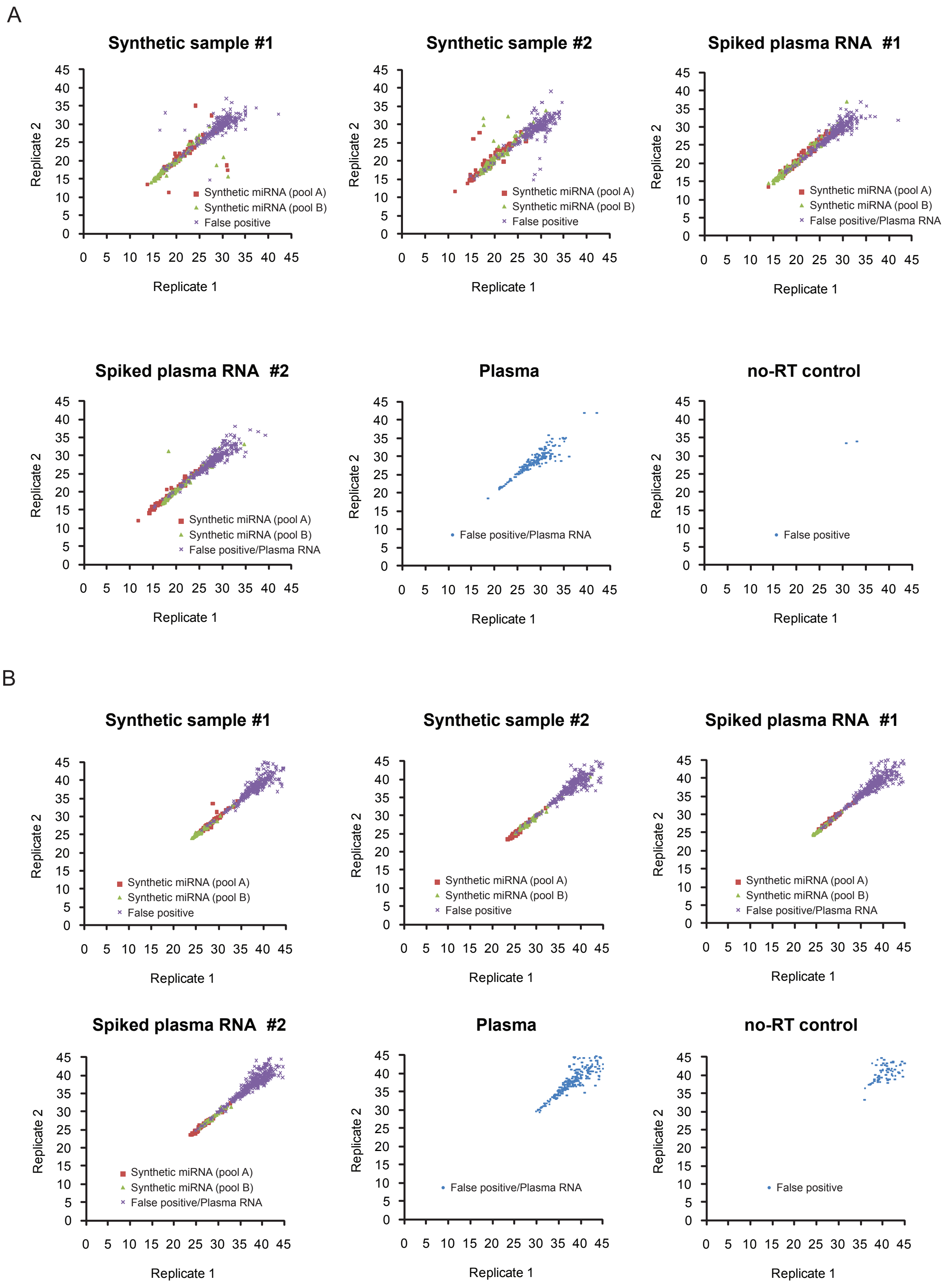

Figure S1 Assessment of reproducibility by scatter plots of the Ct values from replica experiments.

Plotted are the raw Ct values calculated from replica experiments using either the TaqMan platform (A) or the miRCURY platform (B). In order to enable comparison of the individual samples to the no-RT control all assays measured in both replicas were plotted i.e. no Ct detection thresholds were applied.

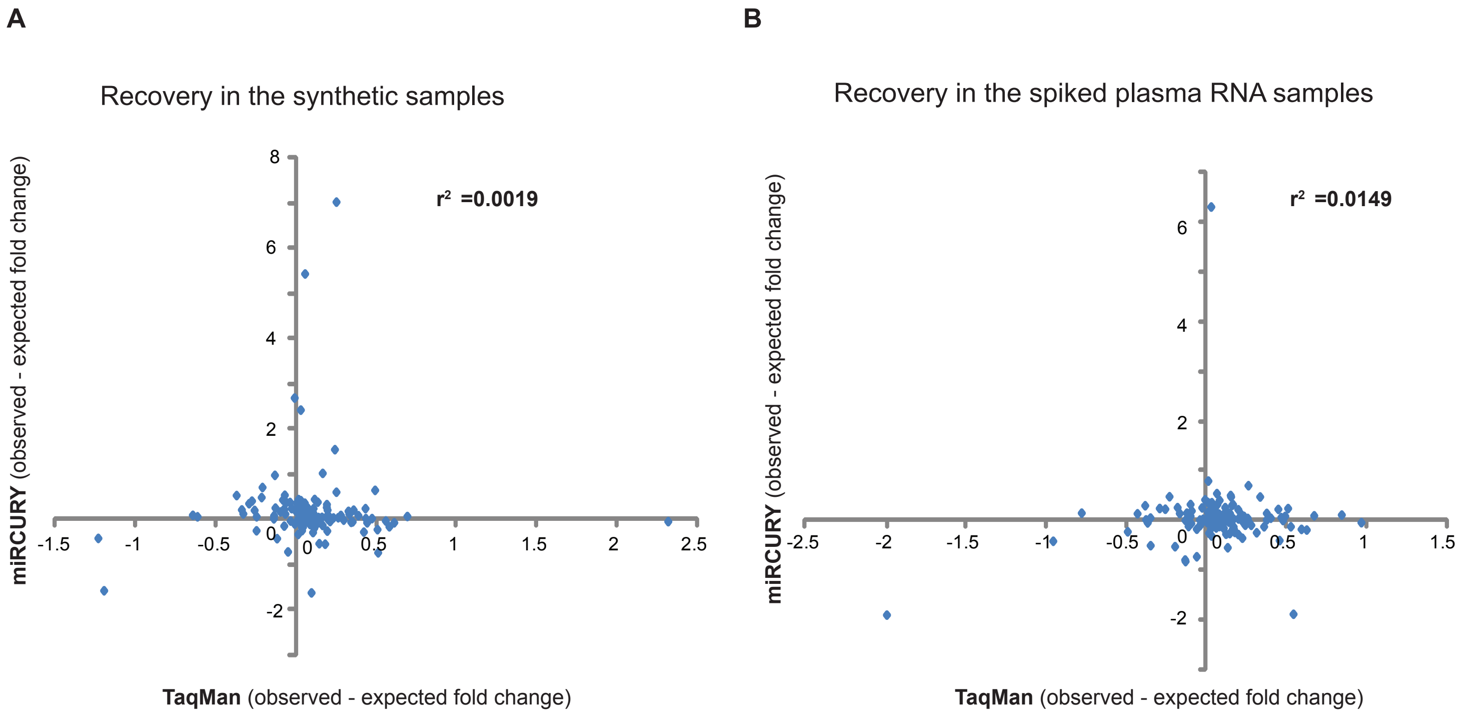

Figure S2 Evaluation of whether the poorly recovered miRNAs of the miRCURY and TaqMan platforms were the same.
Plotted are the differences between the expected and the recovered differences for both platforms. In (A) are plotted the data for the synthetic samples and in (B) for the spiked plasma samples #1 and #2. If the poorly recovered miRNAs of the two platforms were the same, a positive Pearson correlation was expected between the differences of the miRCURY and TaqMan platforms. However, the analyses showed no correlation, indicating that the problematic miRNAs were different for the two platforms.
